# Mesenchymal stromal cell‐derived extracellular vesicles for regenerative therapy and immune modulation: Progress and challenges toward clinical application

**DOI:** 10.1002/sctm.19-0114

**Published:** 2019-08-14

**Authors:** David Allan, Alvin Tieu, Manoj Lalu, Dylan Burger

**Affiliations:** ^1^ Hematology and Blood and Marrow Transplantation The Ottawa Hospital Ottawa Ontario Canada; ^2^ Clinical Epidemiology Ottawa Hospital Research Institute Ottawa Ontario Canada; ^3^ Regenerative Medicine Programs Ottawa Hospital Research Institute Ottawa Ontario Canada; ^4^ Faculty of Medicine University of Ottawa Ottawa Ontario Canada

**Keywords:** extracellular vesicles, immune modulation, mesenchymal stromal cells, preclinical, regenerative therapy

## Abstract

Extracellular vesicles (EVs) derived from mesenchymal stromal cells (MSCs) have emerged as a promising form of regenerative therapy and immune modulation. Fundamental advances in our understanding of MSCs and EVs have allowed these fields to merge and create potential cell‐free therapy options that are cell‐based. EVs contain active cargo including proteins, microRNA, and mRNA species that can impact signaling responses in target cells to modify inflammatory and repair responses. Increasing numbers of preclinical studies in animals with various types of injury models have been published that demonstrate the potential impact of MSC‐EV therapy. Although the emergence of registered clinical protocols suggests translation to clinical application has already begun, several barriers to more widespread clinical adoption remain. In this review, we highlight the progress made in MSC‐derived small EV‐based therapy by summarizing aspects pertaining to the starting material for MSC expansion, EV production, and isolation methods, studies from preclinical models that have established a foundation of knowledge to support translation into the patient setting, and potential barriers to overcome on the path to clinical application.

## Graphical abstract

Cumulative number of published articles investigating the therapeutic efficacy of extracellular vesicles derived from mesenchymal stem cells in preclinical animal models of disease.*Number of articles published in 2018 was extrapolated based on the total number of published articles until May of 2018.

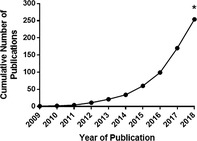

1

**Significance statement**

Mesenchymal stromal cell‐derived extracellular vesicles are a promising cell‐free therapy for regenerative medicine and immune modulation with growing evidence from preclinical animal studies. Bioactive cargo in extracellular vesicles, including proteins, microRNA, and mRNA species, can impact signaling responses in target cells to modify inflammatory and repair responses. Although translation to clinical application has already begun, several barriers to more widespread clinical adoption remain.

## 2 EXTRACELLULAR VESICLES—DEFINITIONS, CLASSIFICATION, HISTORY

Extracellular vesicles (EVs) have a phospholipid bilayer membrane encapsulating cargo from their cell of origin and are released as paracrine effectors to influence target cells as a form of intercellular communication. Mesenchymal stromal cells (MSCs) give rise to stromal and microenvironmental supporting cells found in many tissues of the body and release small EVs as a means of supporting tissue growth such as hematopoiesis in the bone marrow but also modulate inflammatory responses and facilitate tissue repair across a range of tissue injury and organ dysfunction. Since the initial observations that small EVs could facilitate tissue repair, we have learned that most cells release EVs and that signaling molecules such as microRNA, mRNA, proteins, and other small bioactive compounds are packaged as cargo.^1^ A recent classification system has been proposed by the International Society of Extracellular Vesicles that provides a more robust framework for consistent classification than existed previously.^2^ Terms such as exosomes and microvesicles imply knowledge of the origins of the particles but are generally identified by size and surface marker expression. As such, the new guidelines advocate that the terms “small EVs” and “large EVs” be used when origin is not certain. Large EVs (traditionally referred to as microvesicles or microparticles) are often shed from cells under stress following radiation treatment, chemotherapy, ischemic injury, or other stressors.^3^ They may arise from membrane blebbing and are characterized by similar outer cell membrane surface markers as their cell of origin with cytoplasmic contents packaged within their cargo. Large EVs can serve as biomarkers of tissue injury or prognostic markers of adverse events but are rarely considered as potential cell‐derived therapeutic products. Indeed, some evidence suggests that large EVs may counteract the beneficial effects of small EVs.^4^ Small EVs (often referred to as exosomes), in contrast, may arise within endosomes following a programmed cell‐intrinsic pathway that can be altered by external stimuli.^5^ Small EVs can be isolated and used as a therapeutic cell‐derived product for repair of tissue injury or modulation of immune responses and have generated much excitement regarding their potential role in clinical applications.^3^ In this review, we highlight the progress made in MSC‐derived small EV‐based therapy by summarizing aspects pertaining to the starting material for MSC expansion, EV production and isolation methods, studies from preclinical models that have established a foundation of knowledge to support translation into the patient setting, and potential barriers to overcome on the path to clinical application.

## 3 STARTING MATERIAL

**Figure 1:**
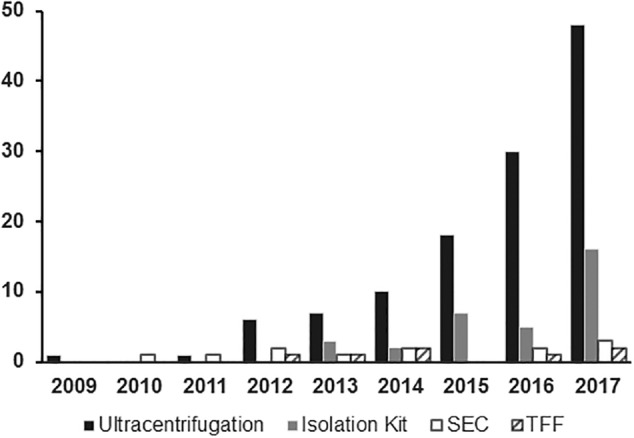
Method of extracellular vesicle isolation. Number of published articles per year subgrouped by method used for isolating extracellular vesicles from mesenchymal stromal cells in preclinical animal models of immune modulation or repair of organ injury

MSCs can be readily expanded from bone marrow, adipose tissue, and placental tissues such as Wharton's jelly or umbilical cord blood. The International Society for Cellular Therapy established minimal criteria to distinguish MSCs from other stromal cells or plastic‐adhering fibroblast‐like cells based on specific cell surface marker expression and retention of multilineage differentiation capacity.^6^ Moreover, the use of early passage MSCs for studies of regenerative therapy has proven essential to avoid issues related to cellular senescence of more extensive MSC passaging.^7^ The development of serum‐free conditions appears encouraging for translation of MSC‐based therapies, and numerous clinical trials of MSCs for regenerative therapy and immune modulation have been conducted using approved media and other reagents.^8^ Heterogeneity, however, in the source of MSCs, dosing, administration schedules, and outcome reporting continues to hamper definitive conclusions regarding the efficacy of MSCs in certain indications such as graft‐versus‐host disease (GVHD).^9^ The observation that conditioned media from MSCs retains much of the therapeutic effects of MSCs themselves has contributed to the development and interest in MSC‐derived small EVs as a therapeutic tool, highlighted by initial studies in acute kidney injury^10^ and myocardial ischemia/reperfusion injury.^11^ Indeed, we recently updated a systematic search of preclinical trials of MSC‐based small EVs in animals with organ injury or immune dysfunction and identified a marked increase in published studies from 17 to 205 between 2013 and 2018 (Figure 1).^12^ Many of these studies used human cells in animal models, and the most common source of cells for the expansion of MSCs is from bone marrow. Most preclinical studies are reporting benefit in one form or another which is encouraging but suggests possible publication bias. A more in‐depth analysis and meta‐analysis of results from these studies is ongoing.

The role that specific ex vivo culture conditions exert on MSC‐derived EV production is interesting and may be a major component of future EV manufacturing and production methods. Hypoxia, for instance, can activate signaling pathways in MSCs to enrich EV content of specific signaling molecules that augment their capacity to promote angiogenesis and facilitate tissue repair in animal models.^13^, ^14^ Hypoxia can also enhance immune modulation by MSCs through increased secretion of indoleamine‐pyrrole 2,3‐dioxygenase (IDO) and increased Treg induction.^15^ Inflammatory conditions, meanwhile, were associated with increased IDO secretion by MSCs, but Treg induction was unchanged.^15^ Other signaling pathways can also be influenced by exogenous signals introduced during the culture phase of EV‐producing cells. Adipose‐derived MSC treated with platelet‐derived growth factor release EVs with enhanced levels of stem cell factor and its ligand, c‐kit, inducing greater angiogenic repair activity,^16^ while exposure of adipose MSCs to basic fibroblast growth factors attenuated the angiogenic activity.^17^ The tissue of origin can also influence the content of MSC‐derived EVs. MSCs derived from bone marrow as compared with cord blood, for example, retain secretome signatures that have a greater influence on bone growth and differentiation, as recently summarized by Rosu‐Myles et al^18^ and others.^19^ Adipose tissue‐derived MSCs, however, appear to maintain similar immune‐modulatory function compared with bone marrow‐derived MSCs.^20^ Combining strategic choices in the starting material to expand MSCs with selective growth conditions may be the optimal approach to influence the therapeutic effects of EVs. Gene transfection and other approaches that target packaging or enrichment of EV production also remain under active study and appear likely to influence EV‐based therapies. MSCs can be easily transduced using lentiviral vectors for overexpressing specific proteins such as angiopoietin 1,^21^ insulin growth factor 1,^22^ and akt^23^ to impact tissue remodeling and organ function, for instance, and other groups have shown enhanced efficacy of MSC‐derived EVs by altering their packaged content with proteins such as CXCR4^24^ and GATA‐binding transcription factor‐4,^25^ or microRNAs such as mIR133b,^26^ miR223,^27^ and miR140.^28^ One important consideration for cell culture conditions is the potential contamination of bovine EVs from serum in growth media.^29^ Hence, the use of serum‐free conditions when isolating cell‐derived EVs are likely necessary to align manufacturing methods with the ever‐increasing stringency of regulatory requirements for accreditation standards.

### 3.1 Isolating and characterizing EVs

**Figure 2:**
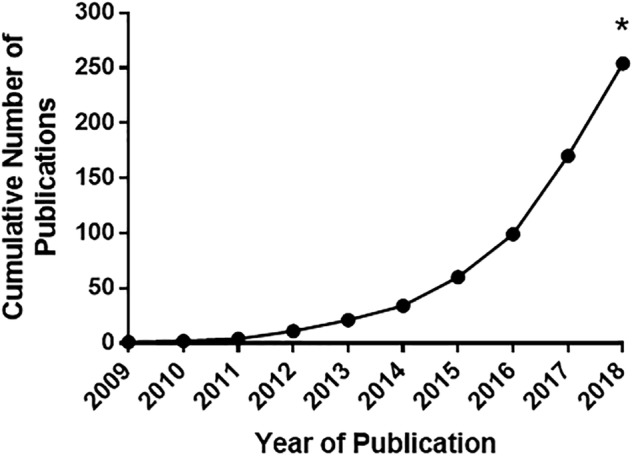
Cumulative number of published articles investigating the therapeutic efficacy of extracellular vesicles (EVs) derived from mesenchymal stem cells in preclinical animal models of disease. The systematic search included Ovid MEDLINE, Ovid MEDLINE in‐process and other nonindexed citations, Embase classic + Embase, and BIOSIS from inception to May 7, 2018, and included studies that addressed interventional in vivo studies using EVs derived from MSCs that were expanded from any tissue source and studied an organ or tissue injury. *Number of articles published in 2018 was extrapolated based on total number of published articles until May of 2018

Initial studies of EV isolation used ultracentrifugation (UC) to separate EVs based on their size. Very high forces in excess of 100 000*g* for prolonged periods are needed to separate the small particles that are <200 nm in size. Unfortunately, UC has been associated with contamination by non‐EV cellular material and reduced yield of RNA compared with density gradient isolation methods which could impact therapeutic efficacy.^30^ More study is needed to understand the impact of different isolation methods on EV content and treatment efficacy. UC remains the most common approach to EV isolation, but is cumbersome and time consuming and not ideally suited to large‐scale production. For these reasons, there has been much interest in developing alternatives. Several kits have been developed for different types of fluids using proprietary polymers to combine and precipitate small EVs. Polymer‐based methods may be used for very small sample volumes and has been associated with biomarker and profiling studies.^31^ The issue of potential contamination with other small particles remains a concern, and impact of polymers on downstream targets that could alter efficacy has been raised in preclinical animal models, which limits enthusiasm. Tangential flow filtration (TFF) has emerged as a relatively easy method of isolating “pure” small EVs that are not complexed with any other molecule and represents a more gentle procedure that appears unlikely to physically alter the EVs.^32^ High yields have been reported^33^ and the potential to apply TFF for larger scale EV production is under active study. Methods reported to date, however, in published preclinical trials of MSC‐EV treatment remain largely based on UC with increasing numbers of studies using TFF in studies published since 2015 (Figure 2). Registered clinical trials that are actively recruiting patients do not provide sufficient technical information, and we will have to await publication of these results or study protocols to gain more insight regarding the clinical relevance of different isolation methods.

### 3.2 Rationale for how MSC‐derived EVs can modulate immune responses and facilitate organ repair

Initial studies that demonstrated how MSCs could modulate immune responses reported a range of cell‐cell contact dependent or independent mechanisms to induce anti‐inflammatory signals and immune tolerance states. In brief, secreted factors such as IL6, IL10, prostaglandin E2 (PGE2), transforming growth factor β (TGFβ), hepatocyte growth factor and IDO have been implicated in affecting target cells of the innate and adaptive immune system.^19^ Collectively, MSC‐derived signaling can inhibit natural killer cell proliferation and activity, reduce B lymphocyte activation, and promote T cells to differentiate into IL17‐producing effector T cells.^34^, ^35^ At the same time, MSC‐associated signaling has been associated with the induction of CD25‐expressing FoxP3‐positive T regulatory cells.^36^ Although some reports have described similar immune‐modulatory responses associated with conditioned media or EVs from cultured MSCs, not all reports are consistent and the mediators of MSC‐EV‐based immune modulation remains incompletely described. Initial reports of MSC‐EVs immune modulation identified several mediators that were similar to MSC‐associated tolerogenic effects, including programmed death ligand‐1, galectin‐1, and TGFβ.^37^ In patients with type 1 diabetes, MSCs expanded from bone marrow samples yielded EVs that contained mRNA and protein of TGFβ and were able to increase the production of TGFβ and PGE2 from T cells treated with MSC‐EVs. Moreover, MSC‐EVs were able to decrease interferon γ (IFNγ) secretion which is associated with downregulation of Th1 responses, increased proportion of Tregs, and reduction in Th17 differentiation.^38^

MSC‐derived EVs have also been implicated in vascular repair following ischemic injury in various animal models. Although mechanisms of vascular repair remain under active study, activation of vascular endothelial growth factor receptors and downstream angiogenesis pathways has been reported^39^ in association with hind limb ischemia repair in a mouse model. MSC‐EVs were also used to ameliorate blood flow recovery, reduce infarct size, and preserve cardiac function in a rat model of myocardial infarction.^40^ Also, small EVs derived from MSCs overexpressing hypoxia‐inducible factor‐1α have increased packaging of Jagged‐1 and can activate Notch‐mediated pathways in target endothelial cells in ischemia‐related diseases.^41^

In a recent study addressing scalable production of MSC‐derived EVs,^42^ profiling of EV cargo was performed and demonstrated robust packaging of immune‐modulatory cytokines including IFN‐γ‐induced protein 10, macrophage inflammatory protein‐1 β, interleukin‐8, the chemokine growth‐regulated oncogene, and tissue inhibitor of metalloproteinase‐1. Moreover, they identified cytokines related to vascular repair including intercellular adhesion molecule‐1, bFGF, and CD105 and microRNA miR210 which is implicated in vascular repair signaling. Starting material, culture conditions and isolation methods appear to impact EV content and potency as demonstrated by a change in cytokine packaging with the addition of angiogenic supplements such as ischemic brain extract.^42^ Assessing the content of EVs will undoubtedly emerge as an important aspect of regulatory oversight related to production methods for MSC‐EVs.

### 3.3 Preclinical studies

In a systematic review of published studies (up to May 2013) of MSC‐derived EVs, we identified 13 controlled preclinical studies addressing MSC‐derived EV therapy in acute kidney injury, myocardial infarction, hind limb ischemia, liver injury, and hypoxic lung injury while four studies addressed the immune‐modulatory effects of MSC‐EVs in models of cancer.^12^ In fact, only half of the studies isolated EVs that were 40‐200 nm, whereas the remainder used EVs that ranged in size up to 1000 nm. Although the majority of studies (79%) provided explicit information regarding the number of animals allocated to treatment or control groups, only a minority (29%) randomly assigned animals and just one study reported on blinding in any aspect of the study.

**Figure 3:**
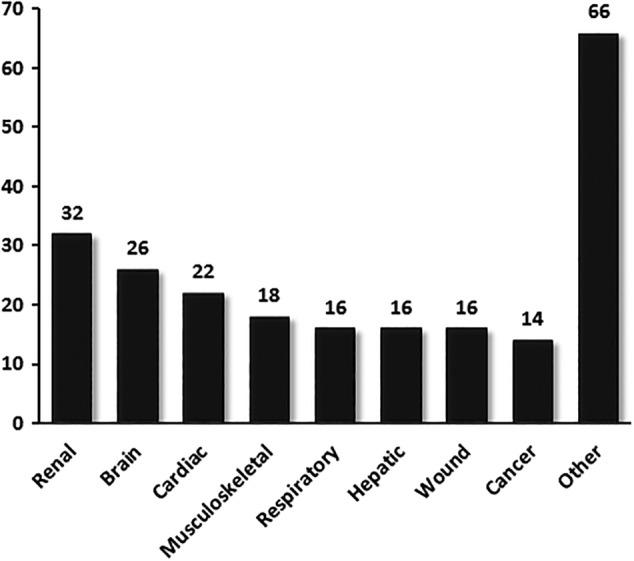
Number of published preclinical studies on mesenchymal stromal cell‐extracellular vesicles by disease domain. Total number of published articles subgrouped by disease domain up to June 1, 2018. Studies investigated extracellular vesicles derived from mesenchymal stem cells as a novel intervention in preclinical animal models. Conditions categorized as “other” include diabetes, ophthalmic conditions, organ transplantation, graft‐versus‐host disease, septic shock, autoimmune conditions, and others

An update to our systematic search was recently performed and reveals a significant increase in the number of publications (Figure 1). A total 205 studies were identified spanning a broader range of organ and tissue injury, including immune‐related conditions such as GVHD, skin allograft rejection, autoimmune conditions and sepsis (Figure 3). Methods of isolating EVs continue to focus mainly on UC, likely due to the ubiquitous availability of equipment and the smaller quantities of EVs needed for small animal studies. Many aspects of experimental approaches, however, remain highly variable from study to study. Parameters including route of administration and dosing of the MSC‐derived EVs have yet to be standardized. Most studies report intravenous delivery of the product (although this can vary in location from jugular vein to tail vein), while many others administer EVs by direct tissue or subcutaneous injection. A number of different dosing strategies are reported, including total absolute protein content of the product, total particle number, dosing by weight of the recipient animal and by amount of EVs produced from a certain quantity of MSCs over a specified time period. Among studies that dosed the EV product from total protein or particle number, the dosage used was highly variable. Future preclinical studies may benefit from dose‐response analyses and biodistribution experiments to gain better pharmacologic insight prior to clinical translation. Although controversial, there has been speculation that MSC therapy may lead to augmented tumorigenesis or ectopic tissue growth. Although some in vitro and in vivo studies have highlighted the potential role of EVs to contribute to tumor growth,^43^, ^44^ the opposite has also been demonstrated where MSC‐derived EVs have led to attenuated tumor size and improved survival in animal models of cancer.^45^, ^46^, ^47^ More detailed studies addressing potential long‐term sequelae of MSC‐derived EVs (whether beneficial or adverse) is needed.

## 4 FOCUS ON CARDIAC REPAIR AND RECOVERY FROM ACUTE KIDNEY INJURY

In our recently published systematic review, the most commonly studied models of organ injury for MSC‐EV therapy were myocardial ischemia and acute kidney injury.^12^ Four studies in cardiac disease^11^, ^48^, ^49^, ^50^ focused chiefly on ischemia/reperfusion injury and all four studies used human fetal tissue‐derived or human embryonic stem cell‐derived MSCs as starting material for EV isolation. All the studies isolated EVs using UC and performed left coronary artery occlusion as their model of ischemia, and all four studies reported significant benefit in terms of perfusion. Safety and longer‐term outcomes were not reported. In our updated search, all cardiac studies again centered around myocardial infarction, either using a permanent ligation or ischemia‐reperfusion model. To date, no clinical studies of cardiac disorders have been published and there are no registered clinical trials on http://clinicaltrials.gov/ addressing cardiac dysfunction, to the best of our knowledge. A forthcoming meta‐analysis of MSC‐EVs as a potential therapy will provide an unbiased estimate of the benefits of this novel cell‐free treatment in the context of cardiac disorders.

We identified a total of six preclinical studies of kidney injury in mice (three studies) or rats (three studies) using human (four studies), mouse (one study) or rat‐derived (one study) MSCs to generate EVs that were isolated by UC (five studies) or density gradient centrifugation (one study).^10^, ^51^, ^52^, ^53^, ^54^, ^55^ Kidney injury was induced by a variety of means including glycerol injection, cisplatin, gentamicin, or partial nephrectomy. All studies reported benefit in biochemical evidence of renal function and histologic improvement of tubular necrosis. A single clinical study of MSC‐derived EV therapy in 40 patients with chronic kidney disease was identified.^56^ Patients receiving EVs had reduced inflammation with increased TGF‐β1 and IL‐10 with reduced tumor necrosis factor (TNF)‐α and improved kidney creatinine clearance compared with placebo‐treated controls.

## 5 FOCUS ON GVHD

Interest in the role of MSC‐EVs in attenuating alloimmune responses may be best exemplified through studies of GVHD following hematopoietic cell transplantation in mice. A total of four controlled preclinical studies of MSC‐EVs addressing the treatment or prevention of GHVD were recently identified in our updated search of literature.^57^, ^58^, ^59^, ^60^ All studies used human MSCs expanded from bone marrow, umbilical cord blood, or embryonic stem cells, and studies isolated EVs using UC or TFF. GVHD was induced by transplanting human leukocyte antigen‐incompatible mouse bone marrow into conditioned recipient mice. The size and characterization of EVs varied and the dosage and administration of EVs was not consistent; however, all studies reported benefit in terms of clinical responses and/or a reduction in the number and severity of mice with GVHD. Moreover, the median survival of treated mice was improved in mice receiving MSC‐EVs compared with controls.

### 5.1 Comparing MSC‐derived EVs with MSCs

In our systematic review published in 2015, we identified four studies addressing acute kidney injury (two studies; 10, 53), hind limb ischemia (one study; 61) and tumor growth (one study; 47) that included MSCs as a control. The remaining studies used placebo as controls and/or used EVs derived from other cell types in the control group. In the studies using MSCs as controls, MSC‐EVs were at least as effective as MSCs and in one case they were more effective, suggesting much of the therapeutic efficacy associated with MSCs can be ascribed to EVs, however, we and the authors recognize that MSCs retain homing abilities that may be superior to EVs and collectively, small numbers of animals were treated in these studies. An updated analysis of control groups used in preclinical studies is forthcoming in our updated systematic review of preclinical studies which may add more insight to this question. On the other hand, MSCs have been associated with potential risks including infusion‐related adverse events such as respiratory compromise and fever, and longer‐term issues such as organ system complications, risk of ectopic tissue formation and malignancy that have required extended follow‐up in clinical trials of MSCs. A meta‐analysis of the safety of MSCs used in clinical trials was reviewed in 2012 and included data from eight randomized controlled trials enrolling a total of 321 patients,^61^ and only fever associated with infusion occurred more often in patients treated with MSCs compared with placebo. More studies with additional patients and longer follow‐up are needed and clinical studies with MSC‐EVs will also require safety and adverse event reporting.

### 5.2 Clinical studies

There are scant reports of MSC‐derived EV administration to patients. In one case report, a patient with steroid‐refractory acute GVHD following allogeneic hematopoietic cell transplantation received MSC‐derived exosomes.^62^ Although a positive response was reported with a reduction in concomitant corticosteroid dosage, the patient died of pneumonia several months after receiving exosome treatment. In a second published report,^56^ 40 patients were enrolled in a controlled clinical trial and 20 patients with chronic kidney disease received two doses of MSC‐EVs given 1 week apart. Treated patients had a 50% improvement in creatinine clearance compared with baseline levels and patients in the placebo group did not experience any change in renal function. Inflammatory markers suggested immune‐modulatory responses in treated patients compared with controls, including increased levels of TGF‐β and IL‐10 and reduced levels of TNF‐α. We are not aware of any other clinical reports, however, we identified four clinical protocols registered with the US National Library of Congress http://clinicaltrials.gov/ (searched February 1, 2019) with at least two of these protocols actively recruiting and/or enrolling patients. In one registered trial (NCT03437759) from a group in China that was first posted in 2018, the investigators are actively recruiting patients with macular holes and significant vision loss who will receive umbilical cord blood MSC‐derived exosomes isolated by UC and will be compared with patients receiving MSCs in a control group. In a second study from Egypt (NCT02138331), the impact of umbilical cord blood‐derived microvesicles on β cell mass in patients with type I diabetes is being examined and continues to enroll patients by invitation. An Iranian team has registered a planned trial (NCT03384433) of intravenous MSC‐derived exosome treatment in patients with acute ischemic stroke using allogeneic MSCs transfected with miR‐124 to enhance neurite remodeling and neurogenesis. Lastly, a study registered by a group at the National Cancer Institute in conjunction with a team at the MD Anderson Cancer Centre in the United States (NCT03608631) outlines a clinical protocol for treatment of patients with metastatic pancreatic cancer harboring a KrasG12D mutation using MSC‐derived exosomes that contain small interference RNA against KrasG12D. Although we are not aware of other published clinical experience with MSC‐derived EVs, it is possible that other investigators have not yet communicated their protocols, results, or experience. With reference to methodological details from these clinical reports, only one of the clinical protocols identified on http://clinicaltrials.gov/ outlined a method for EV isolation (e.g., UC). Furthermore, there is a lack of clear information regarding EV characterization and dosing at this juncture. The final publications from these completed studies should offer more insight.

### 5.3 Translating to the clinic

Preclinical reports of MSC‐EV therapy are highly encouraging across a range of tissue injuries and for modulation of immune responses in disorders such as GVHD and cancer. It appears evident that translational studies in patients are imminent and there are multiple ongoing clinical trials that are actively recruiting patients. Although regulatory approval of EV‐based treatments appears highly feasible and may be simplified in comparison with cell‐based therapies, several possible barriers may contribute to challenges in translating MSC‐EV therapy into the clinical domain. The International Society of Extracellular Vesicles recently published a position paper that provides a thorough discussion of important considerations regarding regulatory and safety issues for EV‐based therapeutics in clinical trials.^5^ Aspects of the starting cellular material remain highly relevant, however, increasing numbers of manufacturing facilities that are accredited by the Foundation for the Accreditation of Cellular Therapy produce MSCs that meet FDA and international regulatory standards. The isolation of EVs from MSCs will require greater standardization and efforts to scale up EV production to levels that can support studies in patients. Characterization of EVs will need to be robust, using methods and approaches that can be validated and approved by regulatory bodies. This will likely include a combination of flow cytometry‐based methods for membrane surface markers and quantitation of EVs for assessment of purity and yield. If MSC‐derived EV products can be used as a third party “off the shelf” product, it may make sense to manufacture and store the product in advance. Storage conditions will need to be optimized and validated to ensure post‐thaw potency using assays that reflect the application being considered, whether it is vascular repair or immune modulation. Any intervention to augment or tailor EV content will require added stringency in terms of validation and approval. In addition to the product, early clinical trials should endeavor to demonstrate safety and tolerability. Efficacy studies will need to include appropriate control groups and standard outcome reporting for each disease domain to facilitate pooling of data for meta‐analysis and development of evidence networks. It has been previously reported that identification of appropriate patient populations, such as in patients with acute kidney injury, can be a challenge for identifying opportunities for translation of preclinical trials.^63^

## 6 SUMMARY

MSC‐derived EVs have been studied increasingly in preclinical models of organ injury and immune disorders and appear as a promising cell‐free regenerative cell‐based therapy for clinical application. More definitive preclinical trials that overcome potential threats to bias by using random allocation of animals to treatment groups and blinding of outcome assessments will accelerate the design of informative initial clinical trials. Translation to clinical studies that meet regulatory approval appears readily feasible. Manufacturing facilities capable of large‐scale EV production will be needed in the near future to support this growing field of study.

## CONFLICT OF INTEREST

D.A. declares consultancy/advisory role with Canadian Blood Services. The remaining authors declare no potential conflict of interest.

## AUTHOR CONTRIBUTIONS

All authors contributed to the manuscript and approved the final version.

7

## Data Availability

Data sharing is not applicable to this article as no new data were created or analyzed in this study.
